# Influence of Lanthanum Precursor on the Activity of Nickel Catalysts in the Mixed-Methane Reforming Process

**DOI:** 10.3390/ijms24020975

**Published:** 2023-01-04

**Authors:** Mateusz Zakrzewski, Oleksandr Shtyka, Jacek Rogowski, Radoslaw Ciesielski, Adam Kedziora, Tomasz Maniecki

**Affiliations:** Department of Chemistry, Institute of General and Ecological Chemistry, Lodz University of Technology, Zeromskiego 116, 90-924 Lodz, Poland

**Keywords:** residual chlorine, lanthanum (III) chloride, nickel catalyst, methane reforming, mechanism, poisoning

## Abstract

This work investigated the influence of the catalytic support precursor on the activity of nickel catalysts 20%Ni/5%La_2_O_3_–95%Al_2_O_3_ in the mixed methane reforming process. The activity tests were carried out at a temperature of 750 °C. The research showed that the catalyst prepared from the precursor containing chloride exhibited very low conversions of methane and carbon dioxide. The poisoned catalyst system before and after the calcination process was subjected to Temperature Programmed Surface Reaction tests to determine whether the thermal treatment causes a decrease in the amount of chlorine in the system. To determine the decomposition temperature of the LaCl_3_ precursor and the nickel chloride NiCl_2_ compound, the samples were analyzed by Thermogravimetry. Finally, the catalytic samples were tested by Time-of-Flight Secondary Ion Mass Spectrometry analysis to confirm the presence of nickel–chlorine bonds on the surface of the catalytic system.

## 1. Introduction

Dry and steam reforming of methane is catalyzed by various common and noble metals such as Ni, Co, Ru, Rh, Pd, and Pt. The order of catalytic activity of metal catalysts supported on Al_2_O_3_ is as follows: Ru > Rh > Ir > Ni > Pt [1]. The absolute values of the activity may vary depending on the process conditions used, while Ni has activity comparable to catalysts prepared with noble metals. Moreover, the activity per volume of the catalyst can be easily enhanced by increasing the content of nickel due to its low production costs. However, Ni compared to noble metals, does not show resistance to carbon deposits and catalyst oxidation [2,3,4,5]. For this reason, many researchers have focused on improving the stability of nickel particles against the sintering and deposition of carbon on their surfaces. Various oxides such as Al_2_O_3_, MgO, TiO_2_, CeO_2_, etc., and binary or ternary oxides have been investigated as supports for methane reforming reactions [6,7,8,9,10,11,12,13,14,15,16,17,18,19,20]. Oxides with low Lewis acid and/or basic sites (e.g., MgO, ZrO_2_, La_2_O_3_) show better catalytic efficiency compared to Al_2_O_3_ or SiO_2_, while some supports (MgO, CeO_2_) also increase the dispersion of nickel particles, thus increasing resistance to sintering and coke formation [12,16,19,20,21]. The addition of La to catalyst support can improve the thermal stability of the catalyst [22]. It should be mentioned that the cause of the catalyst deactivation may also be poisoning. Many catalyst poisons, e.g., ammonia, phosphine, arsenic compounds, sulfur compounds (hydrogen sulfide, sulfides), and chlorine block active surfaces. In addition, poisons can change the structure of the atomic surface of catalysts in a way that reduces the efficiency of the process. Poisoning may be suspected if, during the process, the activity of catalysts drops faster than their specific surface area. However, if the decrease in activity is well correlated with the reduction in surface area, the deactivation can be due to thermal effect, e.g., sintering.

This article investigates the possibility of poisoning the catalyst with chlorine incorporated from the catalytic support precursor. Chlorine may have the effect of reducing or increasing the activity of catalytic systems depending on the process and the type of active phase [23]. Chlorine introduced into the catalytic system may be removed to some extent. According to Cant et al. [24], the oxidation processes are ineffective in removing residual chlorine. In this case, the reduction in catalytic systems is recommended at temperatures above 400 °C. In the case where the chlorine is bounded with alumina, steam is used to remove chlorine [24]. It has also been shown that it is not possible to completely remove chlorine from the Al_2_O_3_ support [25], which may further poison the catalyst by transferring chlorine to the active phase and reducing the number of active centers.

In this work, the influence of the support precursor (LaCl_3_ or La(NO_3_)_3_) on the activity of nickel catalysts in the mixed methane reforming process was investigated [26]. The amount and the type of residual chloride were evaluated using Time-of-Flight Secondary Ion Mass Spectrometry analysis.

## 2. Results and Discussion

### 2.1. Catalytic Activity Measurements

In this work, activity tests were carried out in the mixed methane reforming process to compare the influence of the catalytic support precursor on the activity. Table 1 presents the results of the research on the catalytic activity in the mixed methane reforming process. The tests were carried out using monometallic nickel-based catalyst systems. The prepared catalysts differed in the used precursor of the La_2_O_3_–Al_2_O_3_ support synthesis. In the first case, it was lanthanum (III) nitrate, and on the other hand, it was lanthanum (III) chloride. The conducted studies of catalytic activity show that the catalyst with the nitrate precursor showed higher conversions of methane (71%) and carbon dioxide (68%) while the catalyst obtained using chloride precursor was less active. This is due to the formation of bonds between atomic chlorine and metallic nickel, which consequently leads to a smaller number of active sites on the catalytic surface. This phenomenon will be discussed in later sections of the work.

### 2.2. Temperature-Programmed Hydrogen Chloride Desorption

**Figure 1:**
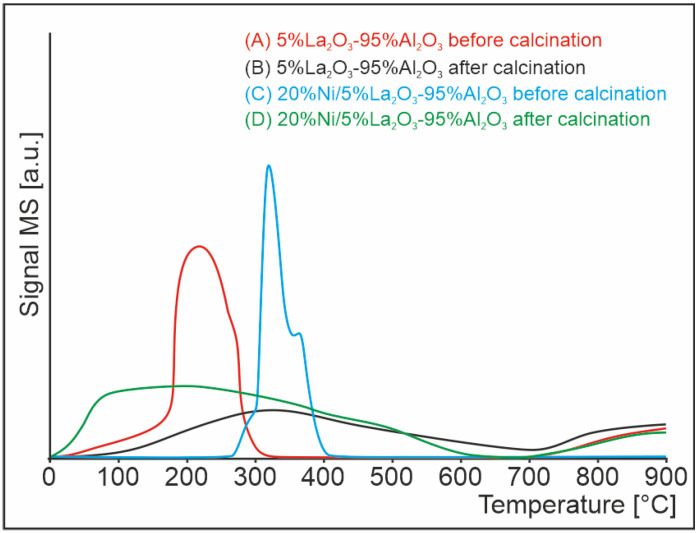
Evolution of hydrogen chloride from (A) 5%La_2_O_3_-95%Al_2_O_3_ before calcination, (B) 5%La_2_O_3_-95%Al_2_O_3_ after calcination, (C) 20%Ni/5%La_2_O_3_-95%Al_2_O_3_ before calcination and (D) 20%Ni/5%La_2_O_3_-95%Al_2_O_3_ after calcination.

In order to verify the presence of chlorine in the catalytic systems prepared using the LaCl_3_ precursor, temperature-programmed surface reaction tests were performed. The reactions were carried out for the 5%La_2_O_3_–95%Al_2_O_3_ supports and 20%Ni/5%La_2_O_3_–95%Al_2_O_3_ catalysts before and after the calcination process. These samples were annealed in a helium atmosphere in the temperature range of 25–900 °C, and the products of the process were recorded with a mass spectrometer. Figure 1 shows four curves representing the amount of chloride ions released in the form of hydrogen chloride from the surface of the tested systems. The first two tests were performed for the La_2_O_3_–Al_2_O_3_ catalyst support before and after calcination marked with red and black curves, respectively. The test performed on the support before the calcination process showed that the chlorine was removed from the surface in two steps. The first low-temperature effect is visible in the temperature range of 40–320 °C, while the second high-temperature effect begins at temperatures above about 730 °C. In the case of the calcined support, two combined peaks related to the evolution of hydrogen chloride in the temperature range from 80 to over 900 °C were observed. Comparing both TPSR profiles, it can be stated that the calcination process did not affect the complete removal of chlorine from the sample. It can be assumed that during the calcination process chlorine is removed from the surface of the support, and the remaining chlorine bound in the mass of the sample may be released from it and re-poison the surface, which is confirmed by the performed tests.

The next TPSR results for the release of hydrogen chloride for the monometallic nickel catalyst before and after the calcination process are shown in Figure 1 by the blue and green curves, respectively. The catalyst before calcination showed two overlapping effects of hydrogen chloride evolution in the temperature range of 280–410 °C. However, the catalyst after calcination (green curve) was characterized only by one wide low-temperature peak in the range of approx. 30–600 °C, and another high-temperature effect above 730 °C. Additionally, for the metal-doped system, it can be seen that the calcination did not completely remove the chlorine from the catalyst sample.

### 2.3. Thermogravimetric TG-DTA Analysis

**Figure 2:**
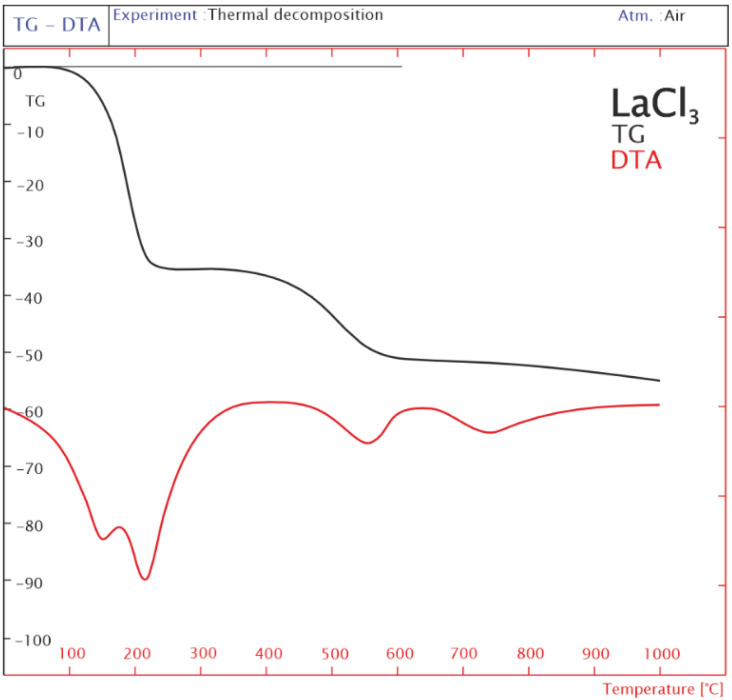
Thermogravimetric analysis of the LaCl_3_ powder.

Chlorine in the form of hydrogen chloride recorded with the mass spectrometer originates from the decomposition of the LaCl_3_ precursor. The TG–DTA studies were carried out to verify the temperature at which lanthanum (III) chloride completely decomposes to the target lanthanum (III) oxide. Figure 2 illustrates DTA and TG traces of the LaCl_3_ sample in an air atmosphere. The results of the TG–DTA measurements of LaCl_3_ showed that the weight loss of the sample included three stages. The TG curve shows rapid weight losses of 34% corresponding to each endothermic peak at 150 °C and 210 °C, respectively in the DTA curve. This weight loss is associated with the dehydration of the sample. The second stage (340–620 °C) is probably connected to the transformation of LaCl_3_ to LaOCl [27]. The last effect at the temperature range 650–1000 °C, visible on the TG curve was related to the decomposition of LaOCl to La_2_O_3_, which is accompanied by the endothermic effect at 740 °C. The tests carried out with the thermogravimetric method show that the LaOCl compound is still present at the reaction temperature of 750 °C. This temperature is too low for the complete decomposition of this compound, so the catalytic system may contain chlorine.

**Figure 3:**
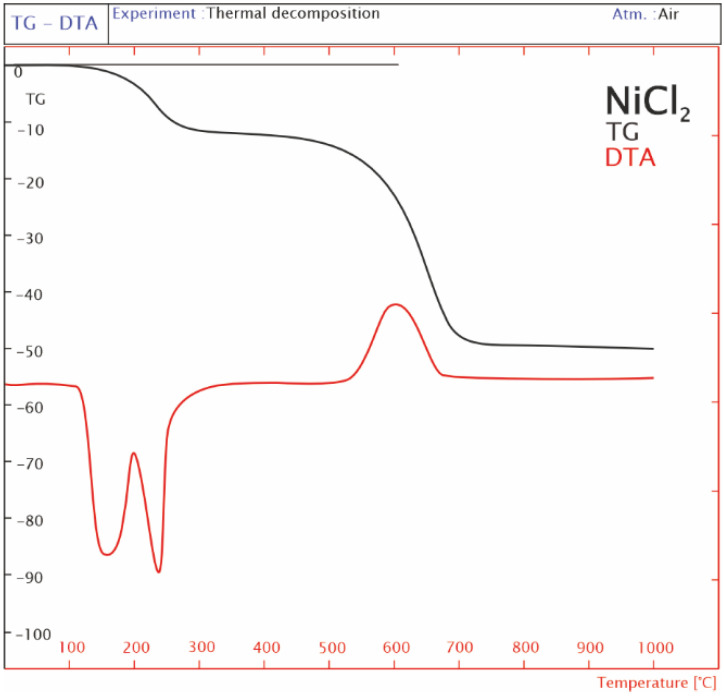
Thermogravimetric analysis of the NiCl_2_ powder.

Chlorine formed as a result of the decomposition of LaCl_3_ can combine with other components included in the structure of the catalytic system 20%Ni/5%La_2_O_3_–95%Al_2_O_3_. One of the elements that can form a bond with chlorine is nickel. These elements can create various combinations, but in this work, nickel (II) chloride was subjected to TG–DTA tests. Figure 3 shows the representative curves of TG and DTA for nickel (II) chloride powder in the temperature range of 25–1000 °C. The result of the TG analysis showed two effects. The first low-temperature effect is located in the temperature range of 100–390 °C. This loss of weight, mapped to a DTA curve in the form of two endothermic peaks, is related to the removal of crystal water from the NiCl_2_ structure. Another effect of the decomposition of the sample is visible in the temperature range of 400–900 °C. This wide mass loss is related to the processes of dehydrochlorination, dichlorination, partial oxidation [28], and the re-crystallization of the formed NiO. The last one is assigned the exothermic effect visible on the DTA curve.

The decomposition reactions of (i) LaCl_3_ × 7H_2_O and (ii) NiCl_2_ × 6H_2_O of the analysis are presented below.

(i) (a) LaCl_3_ × 7H_2_O => LaCl_3_ + 7H_2_O

Figure 2 shows that the water loss in the first stage of the TG-DTA analysis was approx. 35% (17.5 mg out of 50 mg of initial sample weight), which is in good agreement with the theoretical calculations (34% weight loss).

(b) 2LaCl_3_ + O_2_ => 2LaOCl + 2Cl_2_

The analysis of the second stage of LaCl_3_ × 7H_2_O decomposition (Figure 2) shows that the weight loss was 16%, which means that the sample weight lost 8 mg from the initial 50 mg. Appropriate calculations allowed us to conclude that the experimental weight loss of the second stage of the reaction amounted to approx. 25%. The experimental results obtained are in good agreement with the theoretical calculations (27% weight loss).

(c) 2LaOCl + 0.5O_2_ => La_2_O_3_ +Cl_2_

The final decomposition step of the LaCl_3_ × 7H_2_O sample showed a 4% weight loss (50 mg × 4% = 2 mg). The sample after the second stage had a mass of 24.5 mg (50 mg − 50 mg × 51% weight loss = 24.5 mg of solid residue). The theoretical weight loss was 18% and the experiment showed a value of 8%. The results obtained from the TG tests show that the last stage was not completed due to the too-low temperature (1000 °C), which means that the sample still contains chloride forms in the sample mass.

(ii) NiCl_2_ × 6H_2_O => NiCl_2_ + 6H_2_O (theoretical reaction)

Due to the fact that the nickel chloride was dried before the measurements, the water content changed. In Figure 3, the TG curve shows a 12% weight loss in the first stage of the analysis. It is equivalent to the evaporation of 6 mg of water (50 mg × 12% = 6 mg). Therefore, the theoretical water content differs from the results obtained during measurements with a derivatograph. In order to check how much water the new sample of nickel (II) chloride contained, calculations were performed. The result of these considerations is the real formula in the form of NiCl_2_ × H_2_O.

(b) NiCl_2_ × H_2_O => NiCl_2_ + H_2_O (experimental reaction)

(c) 2NiCl_2_ + O_2_ => 2NiO +2Cl_2_

The theoretical weight loss in the decomposition of nickel (II) chloride obtained from the calculations was about 49%. However, the conducted thermogravimetric analysis allowed us to estimate the weight loss at the level of 43%. The difference is not too big, but it can be concluded that up to the temperature of 1000 °C nickel (II) chloride does not completely decompose, which means that chlorine can be present both in the mass and on the surface of the discussed catalytic systems.

Analysis of the decomposition of LaCl_3_ and NiCl_2_ showed that both compounds decompose at high temperatures. This means that the calcined and reduced catalysts still contain some surface and bulk chlorine. The calcination temperature (600 °C) of the La_2_O_3_–Al_2_O_3_ support does not cause lanthanum (III) chloride to decompose completely, which is confirmed by TG–DTA analysis. Lanthanum (III) chloride decomposition products (chloride forms) can move to other components of the catalyst, e.g., aluminum or nickel, and can bond with them, which could be synonymous with poisoning the catalyst.

### 2.4. TOF SIMS Analysis

**Figure 4:**
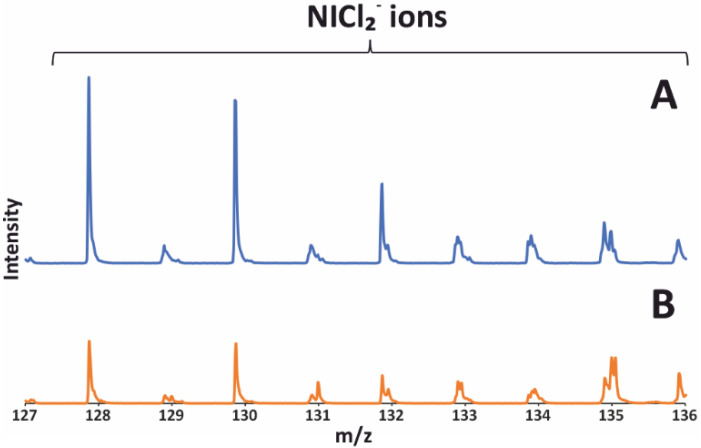
TOF–SIMS spectra of the 20%Ni/5%La_2_O_3_–95%Al_2_O_3_ catalyst (after reduction (**A**) and (**B**) after calcination) presenting peaks of NiCl_2_^−^ ions.

The research results presented above prompted us to use the TOF SIMS technique to investigate the effect of the reduction process on the removal of chlorine from the surface of the catalyst prepared with the use of lanthanum (III) chloride. The TOF-SIMS spectrum of the 20%Ni/5%La_2_O_3_–95%Al_2_O_3_ catalyst reduced at 750 °C is shown in Figure 4A, while the system after calcination is shown in Figure 4B. Both figures clearly show the presence of chlorine on the surface of the studied catalyst. The emission of these ions indicates the presence of the nickel compounds containing chlorine on the surface of the 20%Ni/5%La_2_O_3_–95%Al_2_O_3_ catalyst after reduction and calcination. Moreover, intensities of the peaks of NiCl_2_^-^ ions for the system after the calcination process are much lower than that observed for this catalyst after reduction, which implies that the nickel compounds containing chlorine are effectively formed during the reduction in the catalyst.

## 3. Material and Method

### 3.1. Catalyst Preparation

Alumina oxide was prepared by precipitation method using an aqueous Al(NO_3_)_3_ solution (C = 0.5 mol × dm^−3^). The precipitation process was carried out at the temperature of 80 °C by adding ammonia (as a precipitating agent) to the nitrate solution until the pH of the solution reached values between 9 and 10. The obtained Al(OH)_3_ was aged for 24 h then filtered and washed using deionized water. In the next step, the obtained precipitate was dried at 100 °C for 1 h and calcined in an air atmosphere for 4 h at 500 °C.

La_2_O_3_–Al_2_O_3_ support was obtained using lanthanum (III) nitrate (C = 0.5 mol × dm^−3^) and lanthanum(III) chloride (C = 0.5 mol × dm^−3^). The aqueous nitrate or chloride solutions was added to the previously obtained alumina support. The resulting solutions were left for 24 h followed by evaporation of the solvent. Next, the obtained powder was dried at 100 °C for 1 h, and calcined in air at 600 °C for 4 h. The nominal loading of La_2_O_3_ was 5%.

The monometallic Ni catalysts were prepared by the wet impregnation method. The nickel was introduced on the obtained La_2_O_3_–Al_2_O_3_ support surface using a nickel (II) nitrate (C = 0.5 mol × dm^−3^) precursor. The impregnation process took 12 h. The catalytic material was left for 24 h for impregnation. After the solvent was evaporated, the obtained catalyst was dried at 100 °C and then calcined at 500 °C. The nominal metal content in the obtained catalyst was 20%.

### 3.2. Method and Instruments

The phase transformation of the prepared mixture as a function of temperature was investigated by X-ray diffraction analysis. The diffraction patterns were collected using a PANalytical X’Pert Pro MPD diffractometer in Bragg Brentano reflection geometry (Malvern Panalytical Ltd., Royston, UK). The diffractometer was equipped with a CuK_α_ radiation source (λ = 1.5418 Å). Data were collected in the 2θ range of 5°–80° with a step size of 0.0167° and exposure per step of 27 s. The conducted XRD tests showed the presence of nickel (II) oxide in the catalyst sample after calcination, while the sample after reduction showed the presence of metallic nickel. No other effects were observed in the diffraction pattern. The formed La_2_O_3_ may be non-crystalline and therefore invisible in XRD studies, or its amount may be too small to be visible in the diffraction pattern.

TG-DTA analysis, performed on derivatograph MOM 34-27T (MOM, Budapest, Hungary), was used for the temperature decomposition of the LaCl_3_ and NiCl_2_ in an air atmosphere. The TG-DTA measurements were carried out using a sample with a weight of 20 mg in a temperature range from 25 to 1000 °C at a linear heating rate of 10 °C/min.

The secondary ion mass spectra were recorded using a TOF-SIMS IV mass spectrometer (IONTOF GmbH, Muenster, Germany) equipped with a 25 keV Bi liquid metal primary ion gun and a high mass resolution time of flight mass analyzer. The secondary ion mass spectra were recorded from an approximately 100 μm × 100 μm area of the surface. During measurement, the analyzed area was irradiated with pulses of 25 keV Bi^3+^ ions at a 10 kHz repetition rate and an average ion current of 0.4 pA. The analysis time was 30 s for both negative and positive secondary ions giving an ion dose below the static limit of 1 × 1013 ions cm^−2^.

The temperature-programmed surface reactions (TPSRs) were performed to detect chlorides on the catalytic surface. TPSR measurements were carried out in a quartz reactor using 0.2 g of catalyst in the temperature range of 25–900 °C with a heating rate of 10 °C/min in a helium atmosphere with a volumetric flow of 30 cm^3^/min. The evolution of the gaseous products was analyzed as a function of temperature using a mass spectrometer.

Tests of catalytic activity in the mixed methane reforming reaction were carried out in a flow-type quartz microreactor under atmospheric pressure at 750 °C. The volume ratio of the reaction reagents in each test was as follows: CH_4_:CO_2_:H_2_:H_2_O:Ar = 2:2:1:0.9:1.25. The total flow of the reaction mixture was 100 cm^3^/min. In each test, about 0.4 g of catalyst was placed in the reactor. The catalytic activity was measured after the system was stabilized for 3 h. The gas composition before and after the reaction was measured using gas chromatography with thermal conductivity detectors and two chromatographic columns: (I) Molecular Sieve 5A column (Restek, Bellefonte, PA, USA) and (II) Shin Carbon ST packed column (Restek, Bellefonte, PA, USA).

Methane and carbon dioxide conversions [26,29] were calculated using the following formulas:

$$K_{{\mathrm{CH}}_{4}}=\frac{W_{0{\mathrm{CH}}_{4}}-W_{{\mathrm{iCH}}_{4}}\times \frac{W_{0\mathrm{Ar}}}{W_{\mathrm{iAr}}}}{W_{0{\mathrm{CH}}_{4}}}*100\%$$

$$K_{{\mathrm{CO}}_{2}}=\frac{W_{0{\mathrm{CO}}_{2}}-W_{{\mathrm{iCO}}_{2}}\times \frac{W_{0\mathrm{Ar}}}{W_{\mathrm{iAr}}}}{W_{0{\mathrm{CO}}_{2}}}*100\%$$

where W_i_CH_4_, W_i_CO_2_, and W_i_Ar correspond to the average content of CH_4_, CO_2_, or Ar from three surface measurements originating from the injection of the reaction mixture at a given temperature, and W_0_CH_4_, W_0_CO_2_, and W_0_Ar correspond to the average of the CH_4_, CO_2_, or Ar standard from three surface measurements originating from the injection of the standard mixture.

## 4. Proposed Mechanism of Poisoning the Catalyst with Chlorine

**Figure 5:**
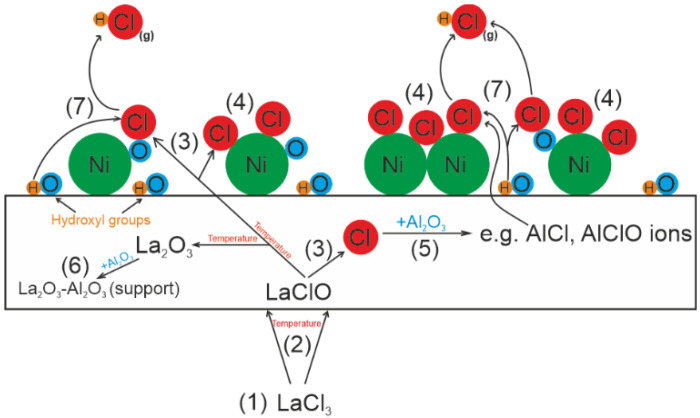
Mechanism of poisoning the catalytic system with chlorine originating from the LaCl_3_ support precursor.

The research presented in this paper allows us to propose the mechanism of poisoning the catalytic surface with chlorine (Figure 5). During the preparation of the 5%La_2_O_3_–95%Al_2_O_3_ catalytic support, chlorine was introduced from the LaCl_3_ precursor (1). Then, the obtained material was calcined in the air atmosphere at a temperature of 600 °C, which according to TG–DTA tests, did not result in the complete decomposition of lanthanum (III) chloride. Due to this process, compound such as LaClO was obtained (2). Chlorine ions formed during the further decomposition of this compound (3) can combine with the active phase Ni (4), giving various types of compounds with Ni. The presence of such compounds in the catalyst I evidenced by NiCl^2−^ ion emission detected by TOF–SIMS. In addition, chlorine ions may also be formed as a result of bond breaking between aluminum and chlorine (5). During the mixed methane reforming process, the Al–Cl bond may break and the chlorine ions formed in this way may diffuse from the bulk of the catalyst to its surface, where it combines with nickel (4).

The final decomposition product of LaCl_3_ is La_2_O_3_ becomes a component of the catalytic support (6). Residual chlorine associated with nickel, aluminum, or lanthanum can be removed from the catalytic system in the form of hydrogen chloride, which is formed by the reaction of chlorine with hydroxyl groups (7) present on the support surface. However, this process does not eliminate chlorine from the catalyst. The temperature of the mixed methane reforming process (750 °C) is also too low to completely remove chlorine from the catalytic bed.

## 5. Conclusions

In the above work, the influence of the catalytic support precursor on the activity of nickel catalyst in the mixed methane reforming process was investigated. TPSR tests carried out in a helium atmosphere showed that in the temperature range of 25–900 °C, chlorine is removed from the system in the form of hydrogen chloride, however, this is not possible with 100% efficiency. The conducted TG–DTA tests showed that LaCl_3_ does not decompose completely up to the temperature of 750 °C, and the resulting chlorine combines with the remaining components of the catalytic system at the stage of preparation, calcination, or reduction. Finally, the TOF–SIMS analysis showed catalysts based on LaCl_3_ precursor after the reduction process, showed significant poisoning of the system with chlorine. The result of this work is the proposed mechanism of surface poisoning with chlorine from the catalytic precursor. Based on the obtained results it cannot be recommended to apply chloride precursors for the preparation of catalytic systems.

## Figures and Tables

**Table 1:** CH_4_ and CO_2_ conversions for monometallic catalyst systems prepared from nitrate and chloride support precursor.

Catalytic System/Support Precursor	Conversion of CH_4_ after 3 h Reaction	Conversion of CO_2_ after 3 h Reaction
20%Ni/5%La_2_O_3_–95%Al_2_O_3_/Lanthanum (III) nitrate	71%	68%
20%Ni/5%La_2_O_3_–95%Al_2_O_3_/Lanthanum (III) chloride	49%	56%

## Data Availability

The data presented in this study are available on request from the corresponding author.
